# Restoration of TGF-*β* signalling reduces tumorigenicity in human lung cancer cells

**DOI:** 10.1038/sj.bjc.6602831

**Published:** 2005-10-25

**Authors:** G Anumanthan, S K Halder, H Osada, T Takahashi, P P Massion, D P Carbone, P K Datta

**Affiliations:** 1 Department of Surgery and Cancer Biology, Division of Surgical Oncology, Vanderbilt-Ingram Cancer Center, Vanderbilt University School of Medicine, 1161, 21st Avenue South, A 3310C MCN, Nashville, TN 37232, USA; 2 Division of Molecular Oncology, Aichi Cancer Center Research Institute, 1-1 Kanokoden, Chikusa-ku, Nagoya 464-8681, Japan; 3 Division of Allergy, Pulmonary and Critical Care Medicine, Department of Medicine, Vanderbilt-Ingram Cancer Center, Vanderbilt University School of Medicine, Nashville, TN 37232, USA; 4 Hematology/Oncology Division, Vanderbilt-Ingram Cancer Center, Vanderbilt University School of Medicine, Nashville, TN 37232, USA

**Keywords:** TGF-*β*1, NSCLC, T*β*RII, RT–PCR, apoptosis, tumorigenicity

## Abstract

Members of the transforming growth factor-*β* (TGF-*β*) family regulate a wide range of biological processes including cell proliferation, migration, differentiation, apoptosis, and extracellular matrix deposition. Resistance to TGF-*β*-mediated tumour suppressor function in human lung cancer may occur through the loss of type II receptor (T*β*RII) expression. In this study, we investigated the expression pattern of T*β*RII in human lung cancer tissues by RT–PCR and Western blot analyses. We observed downregulation of T*β*RII in 30 out of 46 NSCLC samples (65%) by semiquantitative RT–PCR. Western blot analyses with tumour lysates showed reduced expression of T*β*RII in 77% cases. We also determined the effect of T*β*RII expression in lung adenocarcinoma cell line (VMRC-LCD) that is not responsive to TGF-*β* due to lack of T*β*RII expression. Stable expression of T*β*RII in these cells restored TGF-*β*-mediated effects including Smad2/3 and Smad4 complex formation, TGF-*β*-responsive reporter gene activation, inhibition of cell proliferation and increased apoptosis. Clones expressing T*β*RII showed reduced colony formation in soft-agarose assay and significantly reduced tumorigenicity in athymic nude mice. Therefore, these results suggest that reestablishment of TGF-*β* signalling in T*β*RII null cells by stable expression of T*β*RII can reverse malignant behaviour of cells and loss of T*β*RII expression may be involved in lung tumour progression.

Transforming growth factor-*β* (TGF-*β*) belongs to a superfamily of structurally related polypeptides that are involved in various biological processes, including cell growth, differentiation, angiogenesis, apoptosis, and extracellular matrix remodelling (Massague, 1998). The multifunctional effects of TGF-*β* in cellular actions occur by binding to its receptors with intrinsic serine/threonine kinase activity. Sequence comparisons indicate that these receptors fall into two subgroups, designated as type I (T*β*RI) and type II (T*β*RII) serine–threonine kinase receptors. Transforming growth factor-*β* initiate signals by binding to T*β*RII and stabilises the heteromeric complex with T*β*RI, and as a result, T*β*RI is transphosphorylated within a glycine–serine-rich domain (GS-domain). The activated T*β*RI then propagates the signals to intracellular signal mediators including Smads (Engel *et al*, 1998; Massague and Wotton, 2000).

Smad proteins are classified according to their structure and function in signalling by TGF-*β* family members (Attisano and Wrana, 1998). They are characterised by homologous regions at their N- and C-termini known as Mad homology (MH-1 and MH-2) domains, respectively. A divergent linker region separates these domains. Both structural and functional differences provide the basis for a division of the Smads into three groups: receptor regulated (R-Smad), common-mediator, and inhibitory Smads. R-Smads including Smad1, 2, 3, 5, 8, and 9, act as direct substrates of specific type I receptors and are activated by phosphorylation of serine residues at the carboxyl terminus. Thus, Smad2 and Smad3 mediate signalling by TGF-*β* and activin, while Smad1 and presumably Smad5, Smad8, and Smad9 are similarly modified through bone morphogenetic protein (BMP) exposure (Engel *et al*, 1998). Transforming growth factor-*β*/activin receptor-phosphorylated Smads (R-Smads) oligomerise with the common mediator Smad4 (Co-Smad), and after nuclear import, they regulate gene expression positively or negatively by binding to DNA or by interacting with transcription factors (Shi and Massague, 2003). A distinct class of distantly related Smads, including Smad6 and Smad7, has been identified as inhibitors of these signalling pathways. Smad7 forms stable associations with activated type I receptors, thereby preventing R-Smads from binding to and being phosphorylated by these receptors (Hayashi *et al*, 1997; Nakao *et al*, 1997). Thus, Smad7 inhibits TGF-*β*, activin, and BMP signalling. Recently, we have cloned a novel WD40 domain-containing protein called STRAP that associates with both T*β*RI and T*β*RII and is involved in the negative regulation of TGF-*β* signalling (Datta *et al*, 1998). STRAP synergises with Smad7, but not with Smad6, in the inhibition of TGF-*β*-induced transcriptional responses. STRAP associates with Smad7, recruits it from the cytosol to the activated T*β*RI, and stabilises the heteromeric complex, thereby assisting Smad7 in preventing Smad2 and Smad3 activation by the receptor complex (Datta and Moses, 2000). A distinct mechanism of inhibition for Smad6 and its primary role in regulating BMP signals have been proposed (Hata *et al*, 1998; Ishisaki *et al*, 1999). The expression of Smad6 and Smad7 is increased in response to BMP, activin, and TGF-*β*, suggesting a negative feedback of these pathways (Nakao *et al*, 1997; Ishisaki *et al*, 1999).

Perhaps one of the most important biological effects of TGF-*β* is its ability to inhibit proliferation of many cell types, including most epithelial cells (Moses *et al*, 1990). As a result, the mechanism(s) of TGF-*β* growth inhibition has been the subject of intense investigation over the past decade. Transforming growth factor-*β* inhibits progression of cells from G1 into the S phase of the cell cycle (Pietenpol *et al*, 1990; Howe *et al*, 1991). Cell cycle progression is mostly governed by Cdks, which are activated by Cyclins binding and inhibited by the Cdk inhibitors. Although there is not much data linking the known TGF-*β*-signalling pathways with the regulators of cell cycle progression implicated in TGF-*β*-induced growth arrest. Several lines of evidence suggest that Smad signalling is functionally connected directly or indirectly in suppressing the growth of epithelial cells. The primary event that initiates the TGF-*β*-induced growth arrest may be associated with increased expression of p15^INK4B^, p21^Cip1^, and suppression of c-Myc expression. Deregulation of Cdk inhibitors may contribute to TGF-*β* resistance in cancer. Loss of p21^Cip1^ has been observed in advanced breast cancers in association with a poor patient prognosis (Jiang *et al*, 1997). In epithelial cells from the skin, lung, and breast, TGF-*β* rapidly elevates expression of the Cdk4/6 inhibitor p15^INK4B^ (Hannon and Beach, 1994). In keratinocytes, colon and ovarian epithelial cells, TGF-*β* elevates the expression of p21^Cip1^ (Elbendary *et al*, 1994; Datto *et al*, 1995). Smad signalling is required for TGF-*β*-mediated induction of p15^INK4B^ (Feng *et al*, 2000), and p21^Cip1^ (Pardali *et al*, 2000). Transforming growth factor-*β* stimulation of epithelial cells induces the formation of a Smad complex that specifically recognises a TGF-*β*-inhibitory element in the c-Myc promoter, and this response may be critical for TGF-*β*-induced c-Myc downregulation and growth arrest (Chen *et al*, 2001). Dominant-negative Smad3 (Liu *et al*, 1997) or the inhibitory Smad, Smad7 (Itoh *et al*, 1998) blocks TGF-*β*-induced growth inhibition in epithelial cells.

Cell growth is modulated by positive and negative signals, and loss of negative growth constraints may contribute to oncogenic processes. Such perturbations may occur as a consequence of the loss of the tumour suppressor functions of TGF-*β*. One of the physiological roles of the TGF-*β*-signalling pathway is to provide protection against malignant transformation. Loss of TGF-*β* receptor expression has been reported in several tumours, including colon cancer (Markowitz *et al*, 1995), breast cancer (Sun *et al*, 1994), prostrate cancer (Guo and Kyprianou, 1998), and TGF-*β* expression is elevated in these tumours. Resistance to TGF-*β* in lung cancer may occur through several mechanisms that involve functional alteration of signalling molecules. A recent study has demonstrated that most lung cancer cell lines have lost the growth-inhibitory response to TGF-*β* signal (Osada *et al*, 2001). It has been suggested that TGF-*β* resistance stemming from inactivation of T*β*RII could be a multiple process involving both genetic and epigenetic events. Moreover, extensive analyses found that mutations within the coding sequence of the T*β*RII gene are rare in NSCLC. Replication error phenotype, a high incidence of mutation in poly-adenosine (poly-A) tract of the T*β*RII gene was found leading to loss of T*β*RII in colon and gastric cancer (Markowitz *et al*, 1995). Therefore, mutation in the poly-A tract or truncation in the kinase domain of T*β*RII is infrequent in lung cancer. Mutations of Smad2 and Smad4 genes have been found in a limited fraction of lung cancers (5–10%) (Nagatake *et al*, 1996; Uchida *et al*, 1996). Two previous reports have shown (Hougaard *et al*, 1999; Osada *et al*, 2001) that four out of 33 lung cancer cell lines are responsive to TGF-*β*-induced growth inhibition and 29 cell lines are unresponsive to TGF-*β*. However, among these unresponsive cell lines, 21 cell lines show either no expression or weak expression of T*β*RII. Therefore, downregulation of T*β*RII can explain TGF-*β* unresponsiveness in certain fraction of lung cancers. A recent study has suggested that impairment of TGF-*β* signalling may contribute to tumour progression by cell proliferation rather than by modulation of angiogenesis (Park *et al*, 2002). These findings suggest that cancer cells could result in escape from autocrine growth inhibitory effect of TGF-*β* due to the loss of T*β*RII. A correlation between diminished expression of T*β*RII in breast cancer cells and enhanced *in vivo* malignant behaviour has been observed in studies based on patient specimens and an established cell line (Gobbi *et al*, 2000), suggesting that attenuation of the growth inhibitory TGF-*β* autocrine loop in breast cancers worsens clinical outcome. However, little is known about the T*β*RII status in lung cancer and whether restoring TGF-*β* signalling by the introduction of functional TGF-*β* type II receptor alters tumorigenicity in TGF-*β* unresponsive lung cancer cell lines that do not express the type II receptor. In the present study, we have carried out RT–PCR and Western blot analyses with human lung tumour tissues to analyse the expression pattern of T*β*RII for the first time. Out of 46 lung tumour samples analysed by RT–PCR, squamous cell carcinoma (80%), adenocarcinoma (42%), and large cell carcinoma (72%) show downregulation or loss of T*β*RII, and Western blot analyses suggest reduced expression of T*β*RII in 77% of lung tumour samples. We have determined the effects of stable expression of T*β*RII in TGF-*β*-unresponsive human lung tumour-derived VMRC-LCD cells lacking T*β*RII expression. T*β*RII expression restores TGF-*β* downstream signalling as determined by the induction of Smad2/3 and Smad4 complex formation, TGF-*β*-responsive reporter gene activation, inhibition of cell proliferation and induction of apoptosis. We also show that clones expressing T*β*RII reduce colony formation in soft-agarose assay and tumorigenicity in athymic nude mice. Therefore, our data support the notion that impairment of TGF-*β* tumour suppressor function may contribute to lung tumour progression.

## MATERIALS AND METHODS

### Cell culture and biological reagents

Fresh lung tumour and the corresponding normal tissue specimens were collected from 46 patients. These patients had received neither radiotherapy nor chemotherapy before surgery. They underwent pulmonary resection for primary NSCLC at Vanderbilt University School of Medicine Hospital. Informed consent was received and the project was approved by the local Institutional Review Board. VMRC-LCD parental cell line (human lung adenocarcinoma derived) was maintained in RPMI medium supplemented with 10% fetal bovine serum (FBS). Vector control and T*β*RII stable clones were maintained in Geneticin (G418 sulfate; Life Technologies Inc., Carlsbad, CA) selection at 400 *μ*g ml^−1^. All cell lines were maintained at 37°C in presence of 5% CO_2_ in a humidified incubator. Transient transfections were performed using lipofectamine 2000 (Invitrogen, Carlsbad, CA) according to the manufacturer's specifications. Anti-Smad2, anti-Smad3 (Zymed Laboratories Inc., Sanfrancisco, CA), anti-p21^Cip1^, anti-Smad4 antibody (Santa Cruz Biotechnology Inc., Santacruz, CA), antiphospo-Smad2 antibody (Cell Signalling, Beverly, MA) and mouse anti-*β*-actin antibody (Sigma Biochemicals, St Louis, MO) were used in this study. Antiphospho-Smad3 antibody was a generous gift from Dr Edward B. Leof (Mayo Clinic, Rochester, MN). The TGF-*β* receptor kinase inhibitor, SB-431542 was kindly provided by Dr. Nicholas J. Laping from the Glaxo SmithKline, King of Prussia, PA, USA. SB-431542 was also purchased from Tocris Cookson Inc., Ellisville, MO, USA.

### RNA extraction and reverse transcription (RT)-PCR analysis

Total RNA from the tissue specimens was isolated by using Tripure RNA Purification Kit (Roche Applied Science, Indianapolis, IN). RNA quality and concentration were estimated spectrophotometrically at 260 nm. RT–PCR was performed using SUPERSCRIPT II Kit RETROscript™ (Ambion Inc., Austin, TX). The cDNA was synthesised from total RNA (1 *μ*g) using oligo(dT)_15_ primer and reverse transcriptase. The T*β*RII (493 bp) gene fragment was amplified by using the following primers; 5′-gcacgttcagaagtcggtta-3′ (forward) and 5′-gcggtagcagtagaagatga-3′ (reverse). The primer sequences for GAPDH fragment (600 bp) were 5′-ccacccatggcaaattccatggca-3′ (forward) and 5′-tctagacggcaggtcaggtccacc-3′ (reverse). PCR amplification was carried out using 2 *μ*l of the above RT reaction mixture and 1 × PCR buffer, 1.5 mM MgCl_2_, 2 U of Taq DNA polymerase, and 10 nm of each Primer. After initial denaturation at 94°C for 3 min, amplification conditions were as follows: 30 cycles of 94°C for 30 s, 55°C for 30 s, and 72°C for 1 min; followed by a final 5 min extension at 72°C. The RT–PCR products were separated on a 1.5% agarose gel.

### Transcriptional response assay

VMRC-LCD cells were transiently transfected with CMV-*β*-gal, and p3TP-Lux, or (CAGA)_9_ MLP-Luc or p21^Cip1^-Luc reporter plasmids. Transfected cells were incubated in 0.2% FBS in presence or absence of 2 or 5 ng ml^−1^ of TGF-*β*1 for 22 h. Cell lysates were used to measure both luciferase and *β*-gal activities, and the normalised luciferase activity was presented.

### Immunoprecipitation and Western blot analyses

VMRC-LCD cells were serum starved for 2 h and treated in the presence or absence of 5 ng ml^−1^ of TGF-*β* for 90 min. Cells were lysed and equal amount of each protein lysate was incubated with both anti-Smad2 and anti-Smad3 polyclonal antibodies for 2 h at 4°C, followed by incubation with 20 *μ*l of protein G-Sepharose beads (Sigma Biochemicals, St Louis, MO) for 1 h. The immune complexes were analysed by Western blotting with mouse anti-Smad4 antibody. For other Western blots, extracts were prepared from VMRC-LCD cells treated in presence or absence of TGF-*β* at different time intervals indicated in figure legends.

### [^3^H]-Thymidine incorporation assay

VMRC-LCD cells were treated in the presence or absence of TGF-*β*1 for 36 h in 10% FBS medium. 4 *μ*Ci well^−1^ [^3^H]-thymidine (NEN, Boston, MA) was added in each well for an additional 4 h. Cells were then fixed in 10% cold trichloroacetic acid (TCA), washed, and lysed in 0.2 N NaOH. Radioactivity incorporated into TCA-insoluble [^3^H]-thymidine was measured by scintillation counting and presented.

### Cell proliferation assay

VMRC-LCD cells were seeded into 12-well plates. Cells were then treated in the presence or absence of TGF-*β* (0.5 or 5 ng ml^−1^) for a total of 5 days. Transforming growth factor-*β*-containing media was replaced every other day. Cells were counted after 5 days and the average cell numbers from triplicate measurements were plotted.

### Cell death ELISA

VMRC-LCD cells (2 × 10^4^ cells well^−1^) were seeded into 12-well plates and allowed to attach for 20 h. Cells were serum starved for 20 h and then treated in the presence or absence of 5 or 10 ng ml^−1^ of TGF-*β*1 for 24 h. Cells (floating and adherent) were lysed in 200 *μ*l of lysis buffer. Apoptosis in these cells was quantified by using a cell-death detection ELISA kit (Roche Molecular Biochemicals, Laval, Quebec, Canada) according to the manufacturer's instructions. This quantitative sandwich enzyme immunoassay specifically measures the histone region (H1, H2A, H2B, H3, and H4) of mono- and oligonucleosomes that are released during apoptosis. Photometric development was monitored kinetically by reading the plate at 405 nm at 5-min intervals by using a THERMOmax microplate reader (Molecular Devices Corp., Menlo Park, CA, USA). All data points were assessed in triplicate.

### Soft-agarose assay

Soft-agarose assays were performed to compare the clonogenic potential of control and T*β*RII-transfected cells. In all, 5 × 10^4^ cells from each pool were suspended in 1 ml of 0.4% sea plaque agarose containing 10% FBS medium and then plated on the top of 1 ml of semisolidified 0.8% agarose in the same medium in 35 mm plates. For each parental, vector control, and T*β*RII clones, triplicate wells were plated. Plates were incubated for 2 weeks at 37°C in the presence of 5% CO_2_ in a humidified incubator. Colonies grown on soft agarose were counted by automated colony counter.

### Tumorigenicity study

Cells from exponential cultures of VMRC-LCD (5 × 10^6^ cells) were inoculated subcutaneously behind the anterior fore limb of 6-week-old athymic nude mice. Mice were maintained in a pathogen-free facility and tumours were measured two times in a week. Growth curves for xenografts were determined by externally measuring tumours in two dimensions using a slide caliper. Tumour volume was determined from the equation: *V*=(*L* × *W*^2^) × 0.5, where *L* is length and *W* is width of the tumour. Growth curves for tumours were plotted from the mean volume±s.d. of tumours from six mice. The use of these mice in research was approved by Vanderbilt University Institutional Animal Care and Use Committee.

## RESULTS

### Downregulation of T*β*RII in non-small-cell lung cancers (NSCLC)

**Figure 1:**
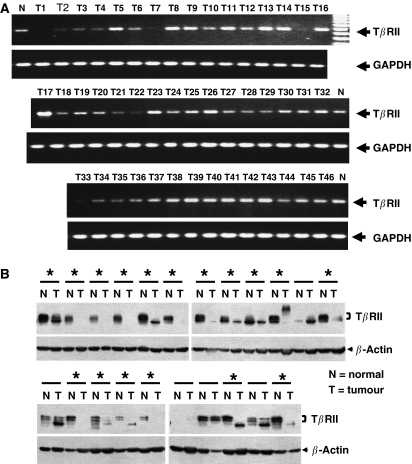
Downregulation of T*β*RII mRNA in lung tumours as determined by RT–PCR. (**A**) Total RNA extracted from human lung tumour tissues were reverse transcribed, and the resulting cDNA was analysed by PCR to test T*β*RII expression. The integrity and equal loading of the RT products was assessed by analysing hGAPDH expression. (**B**) Western blot analysis of T*β*RII protein in lung tumour and corresponding normal lung tissues. Equal amount of lysates were resolved in SDS–PAGE and analysed by Western blotting using antibody against T*β*RII. Equal loading was verified by Western blotting using mouse monoclonal anti-*β*-actin antibody. Tumours with asterisk showed reduced expression of T*β*RII.

Reduced expression of TGF-*β* receptors is known to be associated with unresponsiveness to TGF-*β*-mediated growth inhibitory function and may be involved in tumour progression. To verify whether T*β*RII level is downregulated in human lung tumours, we analysed for T*β*RII expression by RT–PCR using RNA samples from 46 lung tumour specimens (20 squamous cell carcinoma, 19 adeno carcinoma, and seven large cell carcinoma) (Figure 1A). The T*β*RII expression was found to be decreased in 80% of squamous cell carcinoma, 42% adenocarcinoma, and 72% large cell carcinoma. Since we observed reduced T*β*RII mRNA expression in NSCLC, we tested the expression of T*β*RII protein in lysates made from tumour specimens by Western blot analysis. Out of 22 lung tumour samples (including squamous, adeno, and large cell carcinoma) analysed with corresponding control, 17 tumours (77%) showed reduced T*β*RII protein level (Figure 1B). 10 specimens showed almost no expression of T*β*RII. These results suggest that the expression of T*β*RII is reduced in majority of NSCLC patients.

### Stable expression of T*β*RII in VMRC-LCD cells induces Smad2/Smad3 and Smad4 complex formation

**Figure 2:**
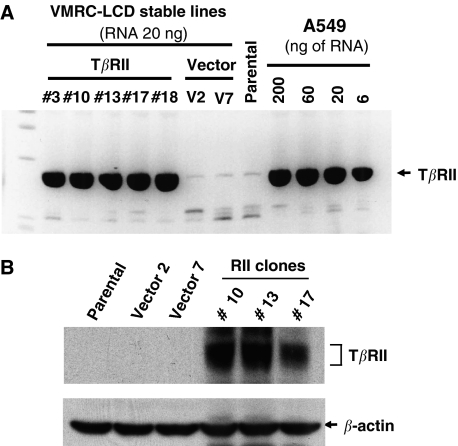
Stable VMRC-LCD cells expressing T*β*RII. (**A**) The VMRC-LCD cells were transfected with T*β*RII-pcDNA3 or empty pcDNA3 vector (Invitrogen) and selected with G418 for 2 weeks to establish the stable clones. In all, 20 ng of total RNA was used for T*β*RII stable VMRC-LCD clones, vector clones and parental cells. Quantitative real-time RT–PCR was performed to determine the relative mRNA expression level of each T*β*RII stable clones. Dilutions of RNA isolated from A549 cells was used as standards for comparison. The stable expression of T*β*RII mRNA in VMRC-LCD clones were compared with the endogenous T*β*RII expression in A549 cells. (**B**) Cell lysates from parental, vector clone, and stable T*β*RII clones were subjected to immunoblotting with anti-T*β*RII antibody. Expression of T*β*RII protein in individual clones is shown. Equal amount of protein loading was verified by immunoblotting the membrane with anti-*β*-actin antibody.

**Figure 3:**
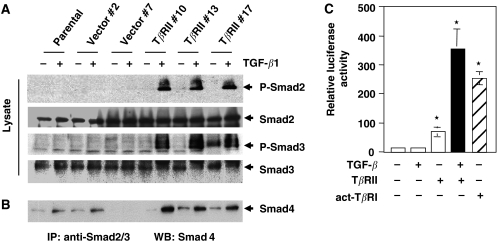
Stable expression of T*β*RII in VMRC-LCD cells restores TGF-*β*-induced phosphorylation of Smad2 and Smad3 and complex formation with Smad4. (**A**) Parental cells, vector control and stable T*β*RII clones were preincubated for 2 h in serum-free medium and then treated with TGF-*β*1 (5 ng ml^−1^) for 90 min. Cell lysates were subjected to immunoblotting with antiphospho Smad2, anti-Smad2, antiphospho Smad3, anti-Smad3, and anti-Smad4 antibodies. Equal amount of protein loading was tested by immunoblotting the membrane with anti-*β*-actin antibody. (**B**) Parental cells, vector control, and stable T*β*RII clones were treated with TGF-*β* as above. Equal amount of cell lysates were subjected to immunoprecipitation with anti-Smad2 and anti-Smad3 polyclonal antibodies and the immunoprecipitates were analysed by immunoblotting with anti-Smad4 antibodies. (**C**) VMRC-LCD parental cells were transiently transfected with (CAGA)_9_ MLP-Luc, and CMV-*β*-gal, T*β*RII or act-T*β*RI (T204D) expression plasmids. Cells were treated with 5 ng ml^−1^ TGF-*β* for 22 h. Luciferase activity was normalised to *β*-gal activity, and the relative luciferase activity was expressed as the mean±s.d. of triplicate measurements. These experiments were repeated at least three times. ^*^P<0.001 for all groups in a multiple comparison test with Bonferroni adjustments after rank transforming the data.

In order to express T*β*RII in VMRC-LCD lung adenocarcinoma cells lacking its expression, wild-type T*β*RII was transfected and cells were selected with G418 to generate stable clonal cell lines. Quantitative RT–PCR was performed to test and to compare the expression of T*β*RII mRNA in stable VMRC-LCD cells and in lung adenocarcinoma cells (A549) (Figure 2A). These data suggest the physiological level of expression of T*β*RII in VMRC-LCD cells. We also tested expression of the protein by Western blot analyses (Figure 2B). Three clones that expressed high levels of T*β*RII (T*β*RII #10, T*β*RII #13 and T*β*RII #17) were selected for further experiments. To test whether overexpressed T*β*RII is functional, we first analysed the phosphorylation of endogenous-positive regulatory Smads, Smad2 and Smad3. Parental cells, two vector control clones, and three T*β*RII clones were treated with TGF-*β* for 90 min and cell lysates were subjected to Western blot analyses by antiphospho-Smad2 and antiphospho-Smad3 antibodies. Phosphorylation of Smad2 and Smad3 (Figure 3A, first and third panel) was found to be increased in T*β*RII stable clones although the expressions of these proteins were unchanged. To test whether stable expression of T*β*RII can restore the complex formation between Smad2/Smad3 and Smad4 *in vivo*, we performed immunoprecipitation experiments after treating T*β*RII clones, vector clones, and parental cells with TGF-*β* for 90 min. Equal amounts of cell lysates were used for immunoprecipitation with both anti-Smad2 and anti-Smad3 antibodies. The immune complexes were analysed by Western blot with anti-Smad4 monoclonal antibody. Transforming growth factor-*β*-induced heteromeric complex formation between Smad2/Smad3 and Smad4 was increased in T*β*RII-expressing clones as compared to parental and vector control clones (Figure 3B). To confirm the TGF-*β* downstream signalling is intact in VMRC-LCD cells, we performed transcriptional assays using TGF-*β*-responsive reporter (CAGA)_9_ MLP-Luc. We observed an increase in transcriptional activity by transfection of T*β*RII and a further strong induction in response to TGF-*β*. Transfection of constitutively active TGF-*β* type I receptor (T204D) (act-T*β*RI) alone induced the reporter activity in VMRC-LCD cells (Figure 3C), because act-T*β*RI does not require TGF-*β* and T*β*RII for downstream signalling. These results suggest that VMRC-LCD cells lack T*β*RII expression and the downstream signalling cascade is intact. Together, stable expression of T*β*RII restores TGF-*β*/Smad signalling in VMRC-LCD cells.

### T*β*RII expression restores TGF-*β*-induced transcriptional responses and p21^Cip1^ expression

**Figure 4:**
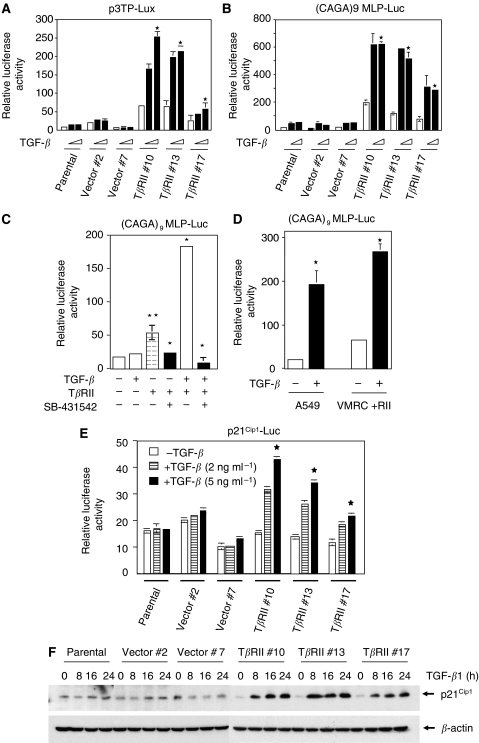
Restoration of TGF-*β*-induced transcriptional responses by stable expression of T*β*RII. Parental, vector control, and T*β*RII stable clones were transiently cotransfected with CMV-*β*-Gal, p3TP-Lux (**A**) or (CAGA)_9_ MLP-Luc (**B**) plasmids. At 20 h after transfection, cells were treated with TGF-*β*1 (2 or 5 ng ml^−1^) for an additional 22 h in low serum (0.2% FBS)-containing medium. Luciferase activity was normalised to *β*-gal activity, and the relative luciferase activity was expressed as the mean±s.d. of triplicate measurements. ^*^P<0.0001 for all groups in (**A**) and (**B**) in a linear mixed effect model on the log-transformed data. (**C**) VMRC-LCD parental cells were transfected with CMV-*β*-Gal, CMV-T*β*RII, and (CAGA)_9_ MLP-Luc plasmids. At 20 h after transfection, cells were treated with 5 ng ml^−1^ TGF-*β* in the presence or absence of SB-431542 (10 *μ*M) for an additional 22 h in low serum (0.2% FBS)-containing medium. Luciferase activity was normalised to *β*-gal activity, and the relative luciferase activity was expressed as the mean±s.d. of triplicate measurements. (**D**) Stable T*β*RII clone and A549 parental cells were transiently cotransfected with CMV-*β*-Gal and (CAGA)_9_ MLP-Luc plasmids. Cells were treated with TGF-*β*1 (5 ng ml^−1^) for 22 h as above. Luciferase activity was normalised to *β*-gal activity, and the relative luciferase activity was expressed as the mean±s.d. of triplicate measurements. ^**^P<0.05, ^*^P<0.005 for all groups in (**C**) and (**D**) in a multiple comparison test with Bonferroni adjustments after rank transforming the data. (**E**) Cells were transiently transfected with CMV-*β*-gal and p21^Cip1^-Luc plasmid and treated with 2 or 5 ng ml^−1^ of TGF-*β*1 for 22 h. Luciferase activity was normalised to *β*-gal activity, and the relative luciferase activity was expressed as the mean±s.d. of triplicate measurements. ^*^P<0.001 for all groups in a linear mixed effect model on the log-transformed data. (**F**) Parental, vector control, and T*β*RII stable clones were serum starved for 16 h and treated with 5 ng ml^−1^ of TGF-*β*1 for different time points. Cell lysates were analysed by Western blotting with anti-p21^Cip1^ antibody (Santa Cruz Biotechnology). Equal amount of protein loading was verified by Western blotting with anti-*β*-actin antibody. Each experiment was repeated three times with similar results.

To explore the functional significance of T*β*RII expression in VMRC-LCD cells, we focused our analyses on TGF-*β*-induced transcriptional responses. We used two TGF-*β*-responsive reporters. The first reporter (CAGA)_9_ MLP-Luc contains multiple Smad3/Smad4-binding CAGA boxes upstream of a minimal adenovirus major late promoter. The second reporter p3TP-Lux containing element from the PAI-1 promoter has both Smad- and Ap1-binding elements. Stable T*β*RII clones showed higher reporter activity in response to TGF-*β*, whereas parental and vector clones did not show any significant response to TGF-*β* (Figure 4A). Similarly, (CAGA)_9_ MLP-Luc reporter activity was strongly induced in response to TGF-*β* in T*β*RII-expressing clones when compared with parental and vector control (Figure 4B). Interestingly, both reporters were induced significantly in T*β*RII clones without TGF-*β* treatment as compared to parental and vector control cells. In an attempt to determine why T*β*RII expression alone induces transcriptional activation, we have performed experiment to block endogenous and exogenous TGF-*β* effect by a TGF-*β* receptor kinase inhibitor (SB-431542) (Halder *et al*, 2005). VMRC-LCD parental cells were transiently cotransfected with TGF-*β*-responsive reporter (CAGA)_9_ MLP-Luc and T*β*RII. Cells were treated with TGF-*β* (5 ng ml^−1^) in the presence or absence of SB-431542 for 22 h. We observed an increase in transcriptional response by T*β*RII alone. This effect was completely blocked by SB-431542 treatment (Figure 4C) suggesting that expression of T*β*RII can restore signalling induced by endogenous TGF-*β*. VMRC-LCD cell line has been shown to secret significant amount of functional TGF-*β* (Halder *et al*, 2005). As expected, this inhibitor also blocks the effect of exogenous TGF-*β*. To compare how well the restoration of TGF-*β* signalling has worked, we performed reporter assay using VMRC-LCD cells stably expressing T*β*RII and TGF-*β* responsive lung adenocarcinoma cells A549 that express T*β*RII. We have observed four- and seven-fold induction in TGF-*β*-induced transcription in VMRC-LCD cells expressing T*β*RII and in A549 cells (Figure 4D), respectively. These results coupled with the Figure 2A suggest that TGF-*β* signalling has been restored in VMRC-LCD cells by the expression of physiological level of functional T*β*RII. To investigate the effect of T*β*RII expression on natural promoter, we performed transient transfection assays with a reporter plasmid containing the luciferase gene under the control of the TGF-*β*-inducible p21^Cip1^ gene promoter. This reporter was induced in T*β*RII clones in response to TGF-*β* but not in parental or vector clones, where the cells do not have T*β*RII expression (Figure 4E). To test whether restoration of TGF-*β* signalling has any effect on endogenous protein expression, we analysed the expression of p21^Cip1^ protein in parental, vector control, and three stable T*β*RII clones in response to TGF-*β* at different time points. The cell lysates were subjected to Western blot analyses using anti-p21^Cip1^ antibody (Figure 4F). Parental and vector clones did not show any induction. However, TGF-*β*-induced p21^Cip1^ protein expression significantly in all three stable T*β*RII clones, and its expression is maintained up to 16 h after the treatment. These results show that VMRC-LCD cells are unresponsive to TGF-*β* due to the lack of T*β*RII expression, and re-expression of T*β*RII makes these cells responsive to TGF-*β*.

### Expression of T*β*RII in VMRC-LCD cells induces TGF-*β*-mediated growth inhibition

**Figure 5:**
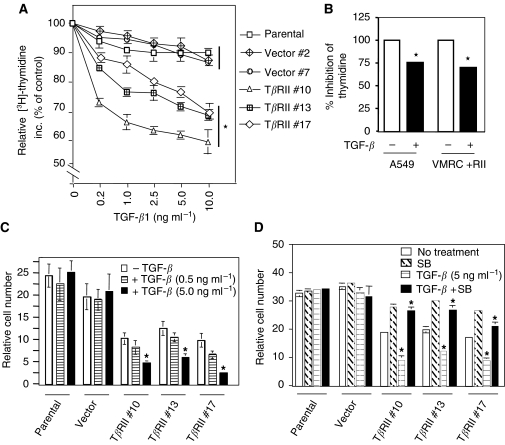
Expression of T*β*RII induces TGF-*β*-mediated growth inhibition. Thymidine Incorporation Assay. (**A**) 30 000 cells well^−1^ of VMRC-LCD in 24-well plate were treated with increasing doses of TGF-*β* in presence of 10% FBS-containing medium for 36 h. 4 *μ*Ci well^−1^ [^3^H]-thymidine (NEN) was added in each well for an additional 4 h. Cells were then fixed, lysed, and the radioactivity incorporated was counted. Radioactivity incorporated without TGF-*β* treatment is considered as 100%, and the results are expressed as the mean±s.d. for triplicate measurements. (**B**) Stable T*β*RII clone and A549 cells were plated as above and treated with TGF-*β* for 36 h. Cells were processed as above and the radioactivity incorporated was counted. Radioactivity incorporated without TGF-*β* treatment is considered as 100%, and the results are expressed as the mean±s.d. for triplicate measurements. ^*^P<0.05 for all groups in (**A**) and (**B**) were compared by Wilcoxon's test. (**C**) Cell counting assay. In all, 8 × 10^3^ cells from parental, vector control, and stable T*β*RII clones were seeded into each well of 12-well plate and then treated with TGF-*β* (0.5 or 5 ng ml^−1^) in 10% FBS-containing medium. Cells were counted after 5 days and plotted. Each data point is expressed as the mean±s.d. of triplicate measurements. Each experiment was repeated three times with similar results. (**D**) VMRC-LCD parental, vector control, and T*β*RII stable clones were treated with 5 ng ml^−1^ TGF*β* in the presence or absence of SB-431542 (10 *μ*M) for 5 days. Cells were counted and the cell numbers were plotted. Individual data points are the mean±s.d. of triplicate determinations. ^*^P<0.005 for all groups in (**C**) and (**D**) in a linear mixed effect model on the log-transformed data.

One of the most important biological effects of TGF-*β* is its ability to inhibit proliferation of many cell types. To determine the effect of T*β*RII expression on TGF-*β*-induced growth inhibition, we first performed [^3^H]-thymidine incorporation assay using control cells and T*β*RII-expressing clones. DNA synthesis in T*β*RII clones is decreased in response to TGF-*β* in a dose-dependent manner. However, we did not observe significant change in thymidine incorporation in parental and vector control cells in response to TGF-*β* (Figure 5A). To compare the growth inhibitory effect of TGF-*β* on DNA synthesis between stable T*β*RII-expressing VMRC-LCD clone and A549 cells with endogenous T*β*RII expression, we performed thymidine incorporation assay. Inhibition in thymidine incorporation by TGF-*β* was 30% in VMRC-LCD stable cells and 25% in A549 cells (Figure 5B). These results suggest that both cell lines has comparable inhibitory effect on thymidine incorporation in response to TGF-*β*. We further tested the effect of T*β*RII expression on cell proliferation by cell counting assays. Parental cells, vector and T*β*RII clones were treated with TGF-*β* and the cells were counted after 5 days. Transforming growth factor-*β* treatment did not affect the growth of both parental and vector control cells, whereas the growth of stable T*β*RII-expressing cells were significantly inhibited by TGF-*β* treatment (Figure 5C). Interestingly, the growth of T*β*RII clones were inhibited in absence of TGF-*β* and this could be due to endogenous TGF-*β* secretion. To confirm the inhibitory role of endogenous TGF-*β* on cell growth in T*β*RII stable cells, we treated the VMRC-LCD parental, vector clones and T*β*RII clones with TRKI (SB-431542) in the presence or absence of TGF-*β*. Growth of parental cells and vector clones was not affected by either TGF-*β* or the inhibitor. However, growth inhibition induced by endogenous or exogenous TGF-*β* was significantly blocked by the inhibitor in stable T*β*RII clones (Figure 5D) suggesting the reestablishment of autocrine endogenous TGF-*β* signalling in these cells. These results suggest that loss of T*β*RII is important for VMRC-LCD cells to be resistant to TGF-*β*-induced growth suppression.

### TGF-*β* induces apoptosis in VMRC-LCD cells expressing T*β*RII

**Figure 6:**
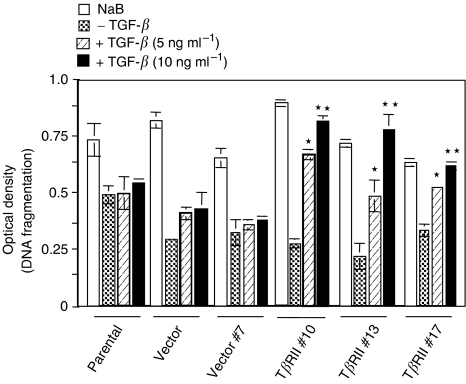
Quantitative cell death ELISA. Parental, vector control, and T*β*RII stable clones were serum starved and treated with either TGF-*β*1 (5 or 10 ng ml^−1^) or 1 *μ*M sodium butyrate for 24 h in serum-free media. Cell lysates were analysed by cell death ELISA as described in Materials and methods. Individual data point is a representative of the mean±s.d. of three individual measurements. Each experiment was repeated three times with similar results. ^*^P<0.0008, ^**^P<0.0001 for all groups in a linear mixed effect model on the log-transformed data.

TGF-*β* is known to induce apoptosis depending on cell types that might contribute to TGF-*β*-mediated tumour suppressor function. To evaluate the role of stable expression of T*β*RII in apoptosis in VMRC-LCD cells, we performed a quantitative cell death ELISA assay using VMRC-LCD cells and stable T*β*RII clones in the presence or absence of TGF-*β*. T*β*RII stable clones showed significant increase in apoptosis in a dose-dependent manner (Figure 6). However, parental and vector control cells did not show any change in apoptosis in response to TGF-*β*. Sodium butyrate induced apoptosis in both control and T*β*RII expressing clones in a similar way. Taken together, we can conclude that expression of T*β*RII in VMRC-LCD cells restore tumour suppressor function of TGF-*β*.

### Stable expression of T*β*RII decreases tumorigenicity of VMRC-LCD cells

**Figure 7:**
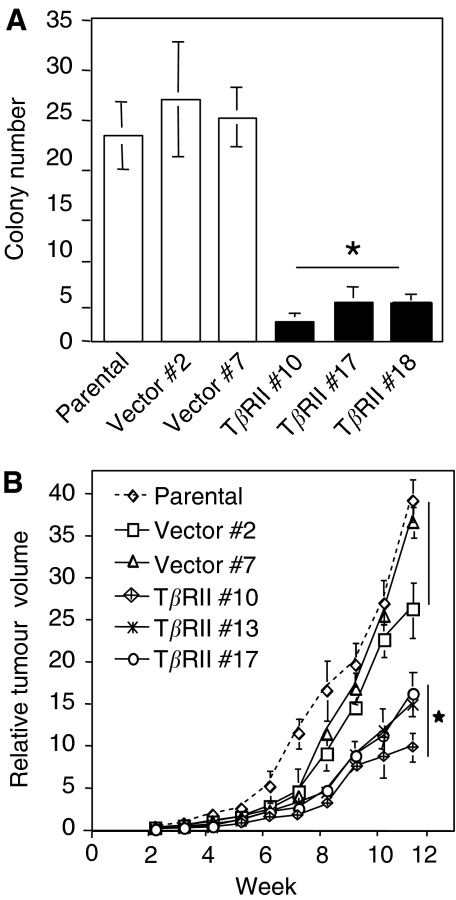
Stable expression of T*β*RII decreases tumorigenicity. (**A**) Restoration of T*β*RII reduces anchorage-independent growth in VMRC-LCD cells. Parental, vector control, and T*β*RII stable clones were plated in soft agarose and incubated for 2 weeks. Colonies were counted by automated colony counter and the data are representative of the mean±s.d. of three values determined from individual plates. ^*^P<0.005 for all groups was compared to control by Wilcoxon test. (**B**) Xenograft growth curves of parental, vector control, and T*β*RII stable clones. Cells (5 × 10^6^) from each pool were subcutaneously injected to the athymic nude mice. Tumours were measured externally on the indicated days in two dimensions using slide calipers. Tumour volume was determined from the equation: *V*=*(L* × *W*^2^) × 0.5, where *L* is length and *W* is width of the tumour. Each data point represents a mean volume±s.e. of six tumours for each group. ^*^P<0.05 for all groups was compared to control by Wilcoxon test.

Loss of TGF-*β* signalling in human tumours is believed to be critical in carcinogenesis. Anchorage-independent growth in semisolid medium and the formation of xenografts in immunocompromised mice are generally considered to be read-outs for assessing the tumorigenicity of human cells. To assess the effect of T*β*RII expression on the malignant properties of VMRC-LCD cells, we compared the ability of the control and T*β*RII stable cells to form colonies in soft agarose. Control clones and T*β*RII stable clones were tested for growth in soft agarose in 35 mm culture plates at 5 × 10^4^ cells well^−1^. After 2 weeks of incubation, colonies were counted by automated colony counter. Significant reduction in colony formation (both size and number) was observed in T*β*RII stable clones when compared with parental and vector cells (Figure 7A). These data suggest that stable expression of T*β*RII decreases the anchorage-independent growth of VMRC-LCD cells. Reduction in cloning efficiency in soft agarose suggested that restoration of TGF-*β* sensitivity might also render VMRC-LCD cells less tumorigenic. To test this hypothesis, we injected exponentially growing cells from parental, vector control and T*β*RII stable clones (5 × l0^6^) subcutaneously in athymic nude mice and followed the progression of xenograft formation. Clones stably expressing T*β*RII consistently formed smaller tumours compared to tumours arising from parental and vector cells (Figure 7B). tumours formed from parental and vector control cells grew substantially faster. Together, these results suggest that overexpression of T*β*RII in a TGF-*β*-resistant lung cancer cell line restored TGF-*β* sensitivity. It is possible that the unresponsiveness to TGF-*β* response in VMRC-LCD cells is merely due to lack of T*β*RII expression and restoration of T*β*RII alone might restore TGF-*β* tumour suppressor function.

## DISCUSSION

The malignant transformation in several types of cancer, including lung cancer, results in a loss of tumour suppressor effects of TGF-*β*. Loss of TGF-*β* response has been shown to be associated with tumour development and/or tumour progression in a number of cancer cell lines (Masui *et al*, 1986; Arteaga *et al*, 1988; Kimchi *et al*, 1988; Hoosein *et al*, 1993; Moustakas *et al*, 1993). However, resistance to TGF-*β* in cancer may occur through several mechanisms such as reduced expression of T*β*RI and/or T*β*RII, mutations or functional inactivation of T*β*RII, inactivating mutations in Smad2 and Smad4 and overexpression of inhibitory proteins including Smad7 (Kleeff *et al*, 1999; Halder *et al*, 2005). In addition, increased production of TGF-*β* by cancer cells during tumour progression favours tumour growth, angiogenesis, and metastasis. Reduced expression of T*β*RII has been implicated as a mechanism for TGF-*β* resistance in both NSCLC and small-cell lung cancer (SCLC). A recent report has suggested that tumours of lung adenocarcinoma from nonsmokers show reduced expression of T*β*RII (Powell *et al*, 2003). In the present study, analyses of RNA and protein from lung tumours demonstrate that T*β*RII expression is reduced or lost in 80% of squamous cell carcinoma, 42% of adenocarcinoma and 72% of large cell carcinoma in comparison to nontumour lesions. We have observed that stable expression of T*β*RII restores TGF-*β*-induced transcription, growth inhibition and apoptosis in a lung adenocarcinoma cell line lacking T*β*RII. As a result these cells become less tumorigenic as determined by soft-agar assay and tumour xenograft studies. These studies suggest that impairment of TGF-*β* signalling through the loss of T*β*RII is involved in lung tumour progression.

Cell growth is modulated by positive and negative signals, and loss of negative growth constraints may contribute to oncogenic processes. Such perturbations may occur as a consequence of the loss of the tumour suppressor functions of TGF-*β*. Reduced expression of T*β*RII has been reported in poorly differentiated lung adeno, and squamous cell carcinoma by immunohistochemical studies (Kang *et al*, 2000). In contrast, another study reported that a reduced expression of TGF-*β*1, T*β*RI and T*β*RII correlated with less lung tumour aggressiveness and a better prognosis (Takanami *et al*, 1997). In these studies the expression levels were determined by immunohistochemistry. However, although we have observed reduced expression of T*β*RII in squamous, adeno and large cell carcinoma by RT–PCR and Western blot, we do not find any direct correlation between expression level and prognosis or differentiation status. Our study is in agreement with the previous studies with lung tumour-derived cell lines that both NSCLC and SCLC cell lines show either no expression or weak expression of T*β*RII in 65–75% cases (Hougaard *et al*, 1999; Osada *et al*, 2001).

One of the physiological roles of the TGF-*β*-signalling pathway is to provide protection against malignant transformation. While TGF-*β* inhibits proliferation of different normal cell types, most tumour cells including gastric, colon, and lung carcinomas are resistant to TGF-*β*-induced growth arrest. As mutations or deletions in receptors or Smads are not common in NSCLC or SCLC, downregulation of the expression of T*β*RII may be a common mechanism for lung tumours to be resistant to TGF-*β* tumour suppressor functions. To test the effect of the loss of T*β*RII expression in lung cancer, we have used lung adenocarcinoma cell line VMRC-LCD that is insensitive to growth inhibitory effects of TGF-*β* and that lacks T*β*RII expression. Our study shows that expression of physiological level of functional T*β*RII restores TGF-*β*-mediated transcription by inducing complex formation between Smad2/3 and Smad4, suggesting that TGF-*β*/Smad-signalling cascade downstream of receptors is intact. A comparable level of TGF-*β*-induced transcriptional response and growth inhibition has been observed in stable T*β*RII-expressing VMRC-LCD clones and A549 cells with endogenous T*β*RII expression. Although these two cell lines are originally derived from lung adenocarcinoma, direct comparison in TGF-*β* signalling is not possible due to different gene expression level and mutational status. Interestingly, T*β*RII-expressing clones are growth inhibited by both endogenous and exogenous TGF-*β* supporting the fact that lung cancer cells could escape from TGF-*β*-induced growth inhibition by losing the expression of T*β*RII. In previous study, we observed that VMRC-LCD cells produce significant amount of TGF-*β* (Halder *et al*, 2005) that may inhibit growth of these clones in an autocrine manner after stable expression of T*β*RII. The effect of endogenous TGF-*β* on growth inhibition in VMRC-LCD cells is further supported by the fact that a TGF-*β* receptor kinase inhibitor specifically blocks this effect in T*β*RII-expressing clones. Although TGF-*β* is known to be a potent growth inhibitor, it also functions as an inducer of apoptosis. Stable expression of T*β*RII in these cells restores TGF-*β*-induced apoptosis. It is possible that loss of TGF-*β*-mediated apoptosis by reduced expression of T*β*RII may be involved in the lung tumour progression. The increased expression and activation of TGF-*β* by tumour cells may induce pro-oncogenic effects that result in the progression of epithelial tumours to the metastatic stage. However, after restoration of TGF-*β* signalling in VMRC-LCD cells we do not observe any change in the tumour-promoting effects of TGF-*β* including cell motility, epithelial to mesenchymal transition, migration and invasion (data not shown).

Loss of TGF-*β* response has been reported to be associated with tumour development and/or progression in a number of cancer cell lines. While some TGF-*β*-resistant cancer cell lines have been shown to retain intact TGF-*β* receptors, majority of the cancer cell lines show the loss of TGF-*β* tumour suppressor function that could be associated with reduced expression of TGF-*β* receptors. We have observed reduced cloning efficiency in soft-agar assay by T*β*RII-expressing clones. In addition, stable expression of T*β*RII in these cells decreases tumorigenicity *in vivo*. Therefore, loss of T*β*RII expression leads to the generation of an aggressive phenotype in lung carcinoma cells and restoration of TGF-*β*-induced tumour suppressor function through the expression of T*β*RII plays an important role in decreasing tumorigenicity.

In conclusion, these data support the notion that TGF-*β* type II receptor plays a critical role in cell proliferation and lung carcinogenesis. The defective expression of T*β*RII may provide an important molecular mechanism in explaining unresponsiveness to TGF-*β* in lung carcinomas. Stable expression of T*β*RII alone plays an important functional role in reducing tumour growth by restoring TGF-*β*-mediated tumour suppressor functions in lung tumour cells that lack T*β*RII. Our study showed that overexpression of T*β*RII restored TGF-*β* sensitivity and reduced the tumour growth. As majority of NSCLC and SCLC cell lines, that are not responsive to TGF-*β*-induced growth inhibition, show weak or no expression of T*β*RII (Hougaard *et al*, 1999; Osada *et al*, 2001), restoration of TGF-*β* signalling through the expression of T*β*RII may be a potential target for chemotherapeutic intervention.
